# Three-dimensional characteristics of nystagmus induced by low frequency in semicircular canals of healthy young people

**DOI:** 10.3389/fnins.2023.1321906

**Published:** 2024-01-04

**Authors:** Xiaobang Huang, Xueqing Zhang, Qiaomei Deng, Shanshan Li, Qiang Liu, Chao Wen, Wei Wang, Taisheng Chen

**Affiliations:** ^1^Department of Otorhinolaryngology Head and Neck Surgery, Tianjin First Central Hospital, Tianjin, China; ^2^Institute of Otolaryngology of Tianjin, Tianjin, China; ^3^Key Laboratory of Auditory Speech and Balance Medicine, Tianjin, China; ^4^Key Medical Discipline of Tianjin (Otolaryngology), Tianjin, China; ^5^Quality Control Centre of Otolaryngology, Tianjin, China

**Keywords:** nystagmus, vertical semicircular canal, rotation test, 3D-VNG, SPV, nystagmus direction

## Abstract

**Objective:**

The study aimed to analyze the three-dimensional characteristics of nystagmus induced by different semicircular canal combinations in healthy young people, and to determine the reference range of nystagmus slow phase velocity (SPV) and its asymmetry.

**Materials and methods:**

Fifty-two healthy volunteers (26 males and 26 females, aged 17–42 years, average 23.52 ± 6.59), were recruited to perform the manual triaxial rotation testing with a 3D-Videonystagmography (3D-VNG) device (VertiGoggles (ZT-VNG-II), Shanghai ZEHNIT Medical Technology Co., Ltd., Shanghai, China) using a 0.3 Hz prompt beat and a 90° amplitude, respectively. The induced nystagmus around the Z-, X-, and Y-axes were recorded in the yaw, pitch, and roll planes. The directions and slow phase velocities of the horizontal, vertical, and torsional components of the induced nystagmus under different semicircular canal combinations (the left lateral and right lateral semicircular canal combination, bilateral anterior semicircular canals, bilateral posterior semicircular canals combination, and the anterior and posterior semicircular canals combination of each ear), as well as their asymmetry, were taken as the observation indexes to analyze the characteristics of the nystagmus vectors of different combinations.

**Results:**

Fifty-two healthy volunteers had no spontaneous nystagmus. The characteristic nystagmus was induced by the same head movement direction in all three axial rotation tests. The SPVs of the left and right nystagmus were 44.45 ± 15.75°/s and 43.79 ± 5.42°/s, respectively, when the subjects’ heads were turned left or right around the Z-axis (yaw). The SPVs of vertically upward and downward nystagmus were 31.67 ± 9.46°/s and 30.01 ± 9.20°/s, respectively, when the subjects’ heads were pitched around the X-axis (pitch). The SPVs of torsional nystagmus, with the upper poles of the eyes twisting slowly to the right and left ears (from the participant’s perspective), were 28.99 ± 9.20°/s and 28.35 ± 8.17°/s, respectively, when the subjects’ heads were turned left or right around the Y-axis (roll). There was no significant difference in the SPVs of nystagmus induced by the same rotation axis in two opposite directions (*p* > 0.05). The reference ranges for the slow phase velocities (SPVs) of nystagmus induced by the triaxial rotation testing were as follows: For the Z-axis (yaw), the SPVs were 13.58–75.32°/s for leftward head rotation and 13.56–74.02°/s for rightward head rotation. For the X-axis (pitch), the SPVs were 13.13–50.21°/s for upward head nystagmus and 11.98–48.04°/s for downward head nystagmus. For the Y-axis (roll), the SPVs were 10.97–47.02°/s for the left-sided head rotation and 12.34–44.35°/s for the right-sided head rotation.

**Conclusion:**

This study clarified the three-dimensional characteristics of nystagmus induced by different semicircular canal combinations in healthy young people. It also established a preliminary reference range of SPVs and SPV asymmetry of nystagmus induced by the vertical semicircular canal. It can further provide a basis for the mechanism of semicircular canal-induced nystagmus and the traceability of nystagmus in patients with otogenic vertigo. It is shown that the portable 3D-VNG eye mask can be used for the manual triaxial rotation testing to achieve the evaluation of the low-frequency angular vestibulo-ocular reflex (aVOR) function of the vertical semicircular canal, which is convenient, efficient, and practical.

## 1 Introduction

Peripheral vestibular spontaneous and induced nystagmus serve as objective signs of asymmetric input from the same plane semicircular canals or different semicircular canal combinations between the two ears. These are crucial for the diagnosis, treatment, rehabilitation, and evaluation of otogenic vertigo. It can be horizontal, horizontally torsional, or vertically torsional (von Brevern et al., 2015; Strupp et al., 2022). The characteristics of peripheral vestibular nystagmus abide by Ewald’s law, but this law is limited to a single semicircular canal effect in animals. Previous studies have also shown that the characteristics of nystagmus in BPPV-Canalolithiasis represent the manifestation of Ewald’s law in a single semicircular canal effect (single canal mode) (Zhang et al., 2021). If two or more semicircular canals or different combinations of semicircular canals get impaired, their detailed nystagmus characteristics (Eggers et al., 2019) are rarely reported. The evaluation of semicircular canal function is a major aspect of the evaluation of vestibular function in patients with vertigo and balance disorders. At present, there are many clinical evaluation methods for the lateral semicircular canal, including the caloric test (0.003 Hz), head shaking test (2 Hz), rotation test (SHAT, 0.01–3 Hz), active rotation test VAT (2–6 Hz), passive rotation test, the video head impulse test (vHIT), (2–5 Hz), etc., involving low-frequency, medium-frequency, and high-frequency functional area detection, with a wide frequency coverage. However, there are few evaluation techniques for the vertical semicircular canal. In 1963, Robinson proposed the magnetic sclera search coil system (Eibenberger et al., 2016), which quickly became the recognized standard for accurately recording eye movement. In 1964, Suzuki et al. (1964) conducted animal experiments, surgically implanting electrodes to stimulate the ampullary nerve of the vertical semicircular canal, and observed the eye movement of four different animals with photographic records. In 1988, Halmagyi and Curthoys (1988) introduced this technology to evaluate the function of the lateral semicircular canal. It was subsequently adopted to evaluate the function of the vertical semicircular canals, thereby evolving into a reliable method for recording the three-dimensional eye movement. In 1996, Fetter and Dichgans (1996) studied the three-dimensional characteristics of spontaneous nystagmus and nystagmus induced by rotation of the semicircular canal in different planes, as well as the dynamic characteristics of the lateral, anterior, and posterior semicircular canals (vestibulo-ocular reflex, VOR) in patients with “vestibular neuritis” by using the magnetic sclera search coil technology. They also conducted vector analysis. Despite its effectiveness, the invasive and costly nature of this technology limited its widespread use from 1988 to 2008 (Halmagyi et al., 2017). In 1988, Halmagyi and Curthoys first reported the HIT technology (Halmagyi and Curthoys, 1988; Curthoys et al., 2023). In 2009, MacDougall (MacDougall et al., 2009) further developed this into a noninvasive variant (vHIT) to detect the lateral semicircular canal abnormalities. By 2013, vHIT could objectively and quantitatively detect high-frequency (2–5 Hz) aVOR injury of the six semicircular canals (Macdougall et al., 2013). Since then, detecting the vertical semicircular canal mainly relies on vHIT and VAT, both of which focus on the high-frequency functional area. In 2001, Iida (Iida et al., 2001) proposed a stimulation method for the vertical semicircular canal by using the head position of 60° backward and 45° left/right inclination. However, this method is only suitable for mechanism exploration, rather than clinical application due to its limited practicality. In the same year, Young (Young et al., 2001) used 3D-VNG (Ulmer, France Synapsys) to conduct the three-dimensional analysis of post-caloric nystagmus in different postures. In 2003, Morita (Morita et al., 2003) explored a method for detecting the vertical semicircular canal abnormalities using different head positions during the traditional low-frequency swivel chair rotation around the vertical axis. However, due to the need for a large swivel chair or invasive technology, as well as the age limitation and difficulty in controlling the head position, the method was deemed impractical. Recently, 3D-VNG has attracted broad attention (Liu et al., 2022). However, it is limited to the three-dimensional nystagmus record and analysis in the caloric test, positional test, and the evaluation of low–medium frequency of aVOR function of the lateral semicircular canal during the rotation tests. In the early stage, our research center initially applied 3D-VNG to analyze the characteristics and mechanism of nystagmus in benign paroxysmal positional vertigo (BPPV) (Liu et al., 2022). This analysis proved that BPPV was the embodiment of Ewald’s law in humans, and can be used as a nystagmus model for the single semicircular canal under single-factor stimulation (Zhang et al., 2021). However, otogenic vertigo diseases usually involve two or more semicircular canals. Therefore, a more comprehensive and effective method is necessary for multi-frequency evaluation of the semicircular canal system. In this study, a portable 3D-VNG eye mask was used to investigate the feasibility of low-frequency aVOR-induced nystagmus in the vertical semicircular canal. The aim was to record and analyze the directions and slow phase velocity (SPV) characteristics of horizontal, vertical, and torsional components of low-frequency aVOR-induced nystagmus of each semicircular canal and different semicircular canal combinations under different axial stimuli via the 3D-VNG, to provide a basis for the mechanism of semicircular canal induced nystagmus and tracing nystagmus in patients with otogenic vertigo. It can also provide a more convenient, efficient, and practical approach for low-frequency measurement technology of all semicircular canals.

## 2 Materials and methods

### 2.1 Participants

From July to September 2023, 52 healthy young volunteers were recruited to record the three-dimensional nystagmus using 3D-Videonystagmography (3D-VNG) in the Department of Otorhinolaryngology, Tianjin Institute of Otorhinolaryngology, and Tianjin Key Laboratory of Auditory Speech and Balance Medicine in Tianjin First Central Hospital. All participants provided informed consent before being included in the study. This study has been approved by the Ethics Committee of Tianjin First Central Hospital.

### 2.2 Methods

**Figure 1:**
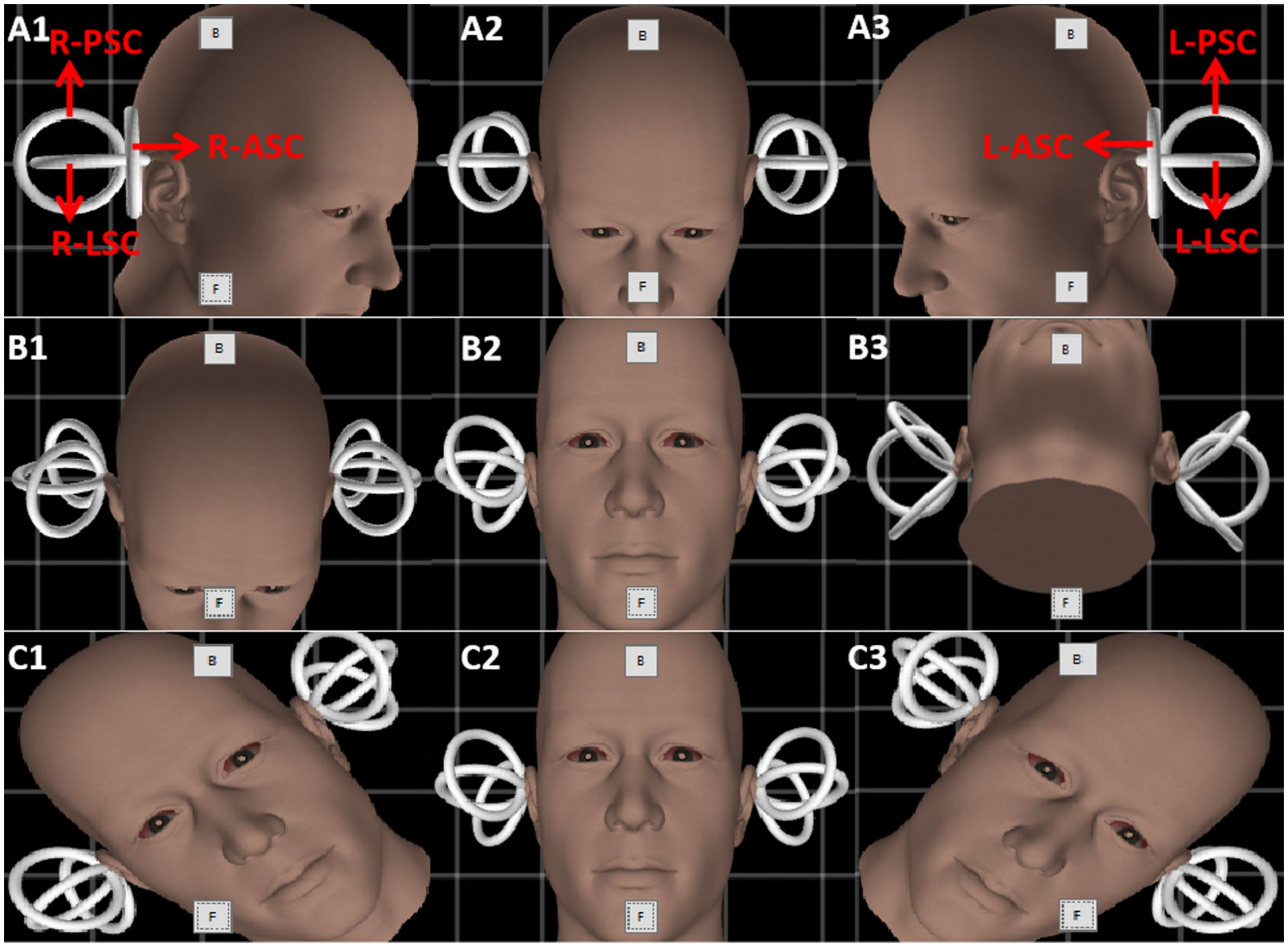
The schematic diagram of the triaxial rotation testing. Gray boxes named “B” and “F” are the buttons to calibrate the gyroscope in every picture. Row **(A–C)** indicate the rotation protocol of three planes (yaw, pitch, and roll). The amplitude of rotation in each direction is 45 degrees. Red arrows depict the six semicircular canals of both inner ears. L-PSC, left-posterior semicircular canal; R-PSC, right-posterior semicircular canal; L-LSC, left-lateral semicircular canal; R-LSC, right-lateral semicircular canal; L-ASC, left-anterior semicircular canal; R-ASC, right-anterior semicircular canal.

There were 52 healthy volunteers (26 males and 26 females, aged 17–42 years, average 23.52 ± 6.59). They all declared no medical history of tinnitus, deafness, dizziness, vertigo, or equilibrium disorders. None of the participants exhibited any cochlear, vestibular, or ophthalmic symptoms or clinical signs, including covert or overt strabismus. Using a 3D-VNG meter (VertiGoggles (ZT-VNG-II), Shanghai ZEHNIT Medical Technology Co., Ltd., Shanghai, China), the same physician performed the manual triaxial rotation testing on volunteers, who were seated upright in the examination chair and wore a 3D-VNG eye mask with two high-resolution and high-frequency cameras recording their binocular movements, respectively. After calibration, the spontaneous nystagmus was recorded first. Thereafter, the triaxial passive rotation test was performed with a prompt beat sound of 0.3 Hz and a total amplitude of 90° rotation (referring to the following study procedure below) (Figure 1). The eye-tracking windows and the eye-tracing curves were used to detect nystagmus, including its direction. Employing different semicircular canal combinations, the characteristics of nystagmus vectors were analyzed by observing the directions, SPVs, and asymmetry of different horizontal, vertical, and torsional components of nystagmus.

### 2.3 Study procedure

The head was turned left and right in the yaw plane around the vertical axis (Z) to stimulate the left and right lateral semicircular canals, respectively (Figure 1, Row A). The head was pitched around the interauricular axis (X) along the pitch plane to stimulate the combination of the anterior semicircular canals of both ears and the combination of the posterior semicircular canals of both ears (double-canal mode), respectively (Figure 1, Row B). The head was tilted to the left and right in the roll plane around the anterior and posterior axis, namely the nasal occipital axis (Y), and the anterior and posterior semicircular canal combination of each ear was stimulated (double-canal mode), respectively (Figure 1, Row C). Then, the induced nystagmus of the passive rotation around the Z, X, and Y axes were recorded.

The asymmetry of induced nystagmus SPV was calculated based on the formulae:

1. SPV asymmetry in horizontal nystagmus induced by the left and right lateral semicircular canals:

- | (L_left_ − L_right_) / (L_left_ + L_right_) | *100%
- = | (SPV_YawL_ − SPV_YawR_) / (SPV_YawL_ + SPV_YawR_) | *100%

1. SPV asymmetry in vertical nystagmus induced by bilateral anterior semicircular and bilateral posterior semicircular canals combination:

- | [(A_left_ + A_right_) − (P_left_ + P_right_)] / [A_left_ + A_right_ + P_left_ + P_right_] | *100%
- **=** | (SPV_PitchA_ − SPV_PitchP_) / (SPV_PitchA_ + SPV_PitchP_) | *100%

1. SPV asymmetry in torsional nystagmus induced by bilateral anterior and posterior semicircular canals combination:

- | [(A_left_ + P_left_) − (A_right_ + P_right_)] / [A_left_ + P_left_ + A_right_ + P_right_] | *100%
- = | [(SPV_RollL_ − SPV_RollR_)] / [SPV_RollL_ + SPV_RollR_] | *100%

### 2.4 Analysis

IBM SPSS Statistics 21 (IBM SPSS, Turkey) was used for statistical analyses. The quantitative data were presented as mean ± SD values and plotted using GraphPad Prism version 10 (GraphPad, San Diego, CA, United States). A *p* value <0.05 was considered statistically significant. The two-sided reference range was ±1.96 SD and the single-sided reference range was ±1.65 SD.

## 3 Results

### 3.1 Demographic characteristics

A total of 52 healthy young volunteers were enrolled, including 26 males and 26 females, aged 17–42 years (average 23.52 ± 6.59). The age range of males was 17–42 years (average 22.12 ± 6.61), while that for the females was 18–37 years (average 24.92 ± 6.39). No significant difference in age was observed between groups (*p* > 0.05).

### 3.2 Characteristics of nystagmus induced by triaxial rotation testing

**Figure 2:**
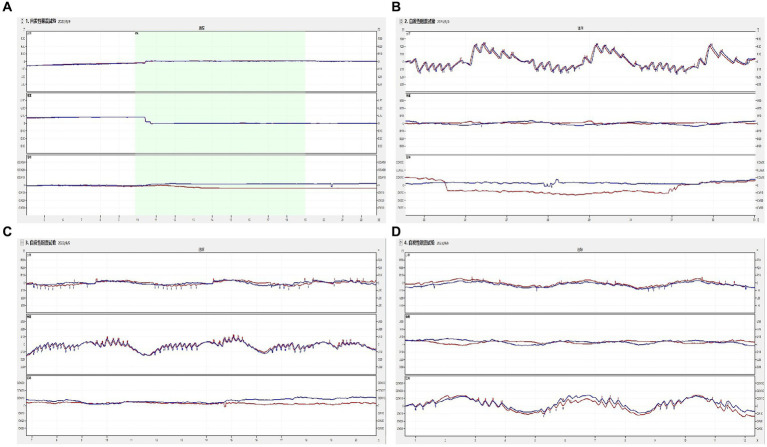
The induced nystagmus by the triaxial rotation testing. The movements of two eyes are displayed from top to bottom in the order of horizontal, vertical, and torsional components, with red lines for the right eye and blue for the left eye. The upward direction of each trace indicates the right, up, and upper pole of the eye beating slowly toward the left ear from the subject’s perspective. **(A)** spontaneous nystagmus. The spontaneous nystagmus (SN) of a participant was negative and remained negative after fixation. **(B)** The head was turned left and right in the yaw plane around the vertical axis (Z). When the physician rotated the subject’s head left to right back and forth at the yaw plane, horizontally left and right nystagmus appeared. **(C)** The head was pitched around the interauricular axis (X) along the pitch plane. When the physician rotated the subject’s head up and down back and forth at the pitch plane, vertically up and down nystagmus appeared. **(D)** The head was rolled to the left and right side around the nasal occipital axis (Y) at the roll plane. When the physician rotated the subject’s head left to right back and forth at the roll plane, torsional (upper pole of the eye beating toward the right/left ear from the subject’s perspective) nystagmus appeared.

**Figure 3:**
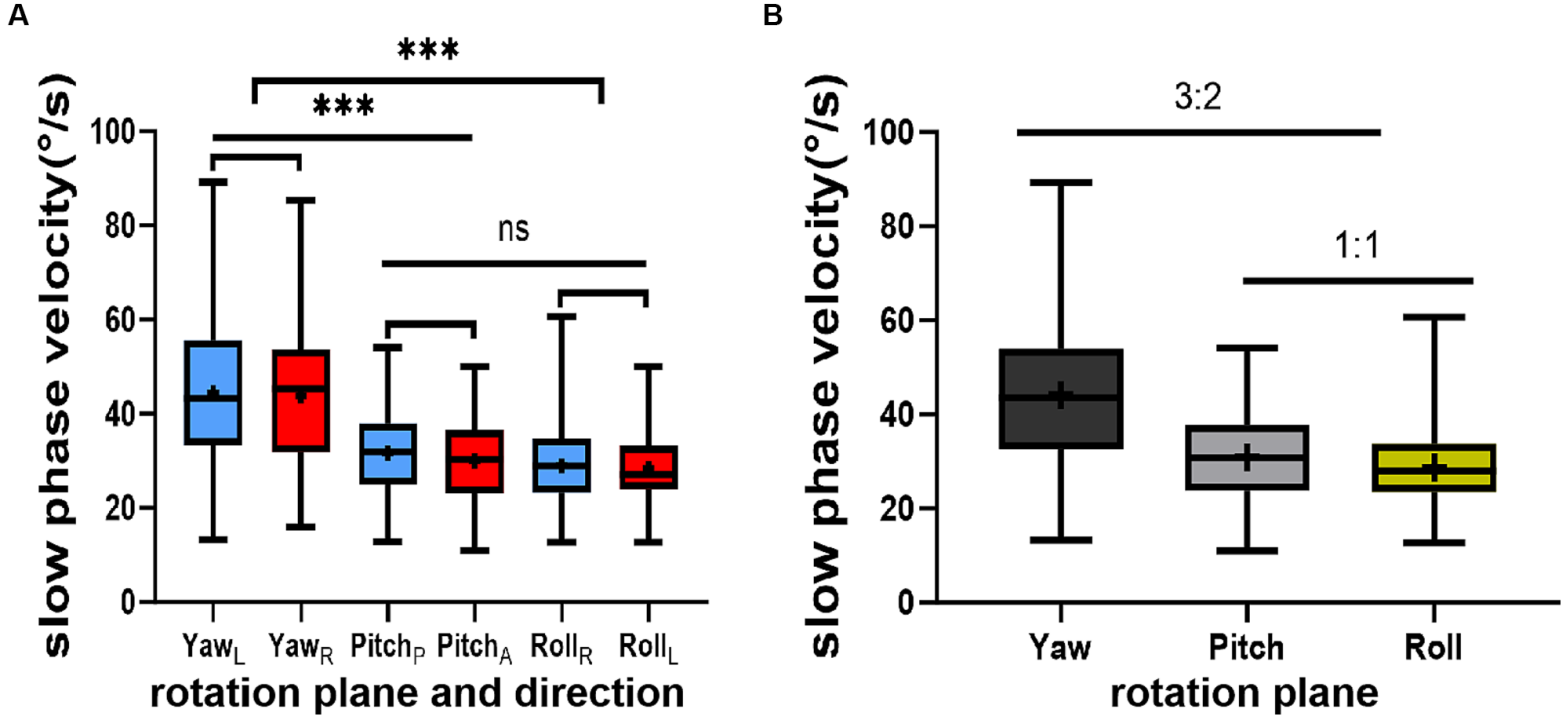
This figure illustrates the results of the triaxial rotation tests. **(A)** depicts the relationship among the slow phase velocities (SPVs) of nystagmus induced by these tests. **(B)** presents the ratios of SPVs of nystagmus induced by the same tests. The direction of nystagmus is indicated as left/right, upward/downward, or Right/Left (the upper pole of the eye beating toward the right/left ear (from the participant’s perspective). The red color represents positive-direction nystagmus or right semicircular canals, while blue signifies negative-direction nystagmus or left semicircular canals. The horizontal (black), vertical (gray), and torsional (yellow) components of nystagmus are also depicted. Statistical significance is denoted as follows: ‘***’ for *p* < 0.001 and ‘ns’ for not significant.

None of the 52 healthy young volunteers exhibited spontaneous nystagmus (Figure 2). The direction of nystagmus induced by triaxial rotation test mirrored the direction of head movement. The SPVs of left-to-right horizontal nystagmus induced by the left and right turning of the head around the Z-axis (yaw) were 44.45 ± 15.75°/s and 43.79 ± 15.42°/s, respectively, with no vertical or torsional components observed. The SPVs of vertical downward-to-upward nystagmus induced by turning the head in the same direction around the X-axis (pitch) were 30.01 ± 9.20°/s and 31.67 ± 9.46°/s, respectively, without obvious horizontal or torsional components. The left and right biases around the Y axis (roll) induced the upper pole of the eye to twist fast toward the subject’s left ear, with the nystagmus SPV recorded as 28.99 ± 9.20°/s, while the SPV for the upper pole of the eye twisting fast to the subject’s right ear was 28.35 ± 8.17°/s, accompanied by a slight horizontal component and no obvious vertical component. The average SPVs of the two-directional nystagmus induced by rotation around the Z-axis (yaw), X-axis (pitch), and Y-axis (roll) were 44.12 ± 15.51°/s, 30.84 ± 9.32°/s, and 28.67 ± 8.66°/s, respectively. A significant difference was observed in the average SPVs of the nystagmus induced by rotation around the Z-axis (yaw) compared with those induced by rotation around the X-axis (pitch) and Y-axis (roll) (*p <* 0.001) (Figure 3A). Among them, the SPV of the three-directional nystagmus induced by rotation around the Z-axis (yaw) was the largest, with the ratio of SPV_Yaw_: SPV_Pitch_ and SPV_Yaw_: SPV_Roll_ of about 3:2, while that of SPV_Pitch_: SPV_Roll_ of about 1:1 (Figure 3B). No significant difference was found in the SPVs of nystagmus induced in different genders and between the aforementioned groups (*p* > 0.05). Similarly, there was no significant difference in the SPVs of the two-directional nystagmus induced by rotating around the same axis (*p* > 0.05).

The reference ranges for SPVs of nystagmus induced by the triaxial rotation testing were as follows:

- For the left-directional rotation around the Z-axis (yaw): (13.58–75.32) °/s.
- For the right-directional rotation around the Z-axis (yaw): (13.57–74.02) °/s.
- For the up-directional rotation around the X-axis (pitch): (13.13–50.21) °/s.
- For the down-directional rotation around the X-axis (pitch): (11.98–48.04) °/s.
- For the left-directional rotation around the Y-axis (roll): (10.97–47.02) °/s.
- For the right-directional rotation around the Y-axis (roll): (12.34–44.35) °/s.

The reference ranges of SPVs of horizontal nystagmus induced by rotation around the Z-axis (yaw), vertical nystagmus induced by rotation around the X-axis (pitch), and rotational nystagmus induced by rotation around the Y-axis (roll) were 13.72–74.52°/s, 12.57–49.11°/s and 11.7–45.64°/s, respectively (Table 1).

**Table 1:** The direction and SPV of induced nystagmus in 52 subjects.

Rotation plane	Yaw		Pitch		Roll	
Direction	Left	Right	Upward	Downward	Right	Left
Sex (M:F)	1:1	1:1	1:1	1:1	1:1	1:1
M* SPV (°/s)	42.81 ± 13.63	43.31 ± 12.28	31.78 ± 8.27	30.72 ± 7.53	28.11 ± 6.80	27.80 ± 7.41
F* SPV (°/s)	46.08 ± 17.73	44.27 ± 18.27	31.56 ± 10.68	29.30 ± 10.72	29.87 ± 11.17	28.89 ± 8.97
Both SPV (°/s)	44.45 ± 15.75	43.79 ± 15.42	31.67 ± 9.46	30.01 ± 9.20	28.99 ± 9.20	28.35 ± 8.17
RV_SPV_ (°/s)	(13.58, 75.32)	(13.57, 74.02)	(13.13, 50.21)	(11.98, 48.04)	(10.97, 47.02)	(12.34, 44.35)
SPV (°/s)	44.12 ± 15.51		30.84 ± 9.32		28.67 ± 8.66	
RV_SPV_ (°/s)	(13.72–74.52)		(12.57–49.11)		(11.7–45.64)	

Both, male and female; M, male; F, female; ^*^p > 0.05; left/right, upward/downward, right/left (upper pole of the eye beating toward the right/left ear (from the participant’s perspective), the direction of nystagmus; SPV, slow phase velocity; RV, reference value of slow phase velocity.

### 3.3 The asymmetry of induced nystagmus SPVs and its reference range by triaxial rotation testing

SPV asymmetry for the nystagmus induced by the triaxial rotation testing was 11.8 ± 9.1% for yaw, 15.1 ± 13.0% for pitch, and 10.8 ± 7.8% for roll, respectively (Table 2).

**Table 2:** The induced nystagmus SPV asymmetry of multiaxial rotation test and its reference value in 52 subjects.

Rotation plane	Yaw	Pitch	Roll	*P* – value
SPV asymmetry (%)	11.8 ± 9.1	15.1 ± 13.0	10.8 ± 7.8	0.140**
RV_SPV Asymmetry_ (%)	(0, 26.8)	(0, 36.6)	(0, 23.7)	/

**p > 0.05; SPV, slow phase velocity; RV_SPV Asymmetry_, reference value of slow phase velocity asymmetry; ‘/’, no value.

## 4 Discussion

### 4.1 Three-dimensional characteristics and clinical significance of the low-frequency induced nystagmus of each semicircular canal in healthy young people

The detection of vertical semicircular canal function has posed a significant challenge in clinical vestibular medicine. The traditional sine harmonic acceleration (SHA) around the vertical axis is limited to detecting the aVOR function in the low-frequency region of the lateral semicircular canal. Although vHIT can detect all six semicircular canals, it only detects the aVOR function in the high-frequency region of each semicircular canal (Liu et al., 2023; Ramaioli et al., 2023). Despite many studies (Baloh et al., 1986; Iida et al., 2001; Young et al., 2001) have explored methods to detect the low-frequency region of the vertical semicircular canal, their clinical applicability remains limited due to the high cost, invasiveness, age restrictions, and difficulties of head position control.

In this study, a 3D-VNG eye mask was used to perform the manual triaxial rotation testing focusing on the vertical semicircular canal. The results showed no statistical significance when comparing SPVs of nystagmus induced by two opposite directions of the same rotation. This suggests that this portable manual triaxial rotation testing could consistently stimulate nystagmus to the same extent in two opposite directions of the same rotation axis, with equivalent nystagmus SPVs. This aligns with the characteristics of vestibular function examination, where equal physiological stimulation can induce an equal effect, which can provide a reliable reference for future studies on the mechanism and location of damage in the different vertical semicircular canals at low frequencies. This study also showed that the type of nystagmus is contingent on the plane of head movement: the horizontal nystagmus was primarily observed with left and right head turns in the yaw plane, the vertical nystagmus with head tilts in the pitch plane, and the torsional nystagmus (accompanied with a slight horizontal component) mainly with left and right head turns in the roll plane. The SPVs of induced nystagmus in the yaw plane were the largest, followed by the pitch plane, while the roll plane was the smallest. The SPV ratio for yaw plane to pitch plane-induced nystagmus was approximately 3:2, while that for pitch plane to roll plane-induced nystagmus was approximately 1:1. The reasons for the mild horizontal component of nystagmus induced by the left and right head deviation in the roll plane and the unequal SPV in the three planes may be due to off-axis stimulation of the vertical semicircular canal. This aligns with reports by Benson et al. (1989) and Allred and Clark (2023) suggesting a higher perception threshold for the vertical semicircular canal than that for the lateral semicircular canal. In 1979 and 2023, it was reported that when the rotation axis is not parallel to the gravitational acceleration vector, the effect of the velocity storage mechanism diminishes or disappears (Raphan et al., 1979; Allred and Clark, 2023). This characteristic was also observed in this study. In 1988, Lafortune et al. (1988) studied the effect of otoliths organ-mediated activity induced by triaxial active head position changes coupled with human horizontal velocity storage on optokinetic afternystagmus (OKAN) in 16 subjects. They found that OKAN was inhibited along the pitch or roll plane but enhanced along the yaw plane, suggesting a potential correlation.

In 2003, Morita (Morita et al., 2003) stimulated the vertical semicircular canal by tilting the head back 60° and rotating it 45° from the sagittal plane to either side. The findings revealed that the vertical semicircular canal function was less affected by the velocity storage mechanism than the lateral semicircular canal function. These results are consistent with previous research (Liu et al., 2022), and the relationship between nystagmus SPVs (3:2 and 1:1) is consistent with the physiological characteristics of the semicircular canals. In addition, this study established the reference ranges for SPVs induced by the triaxial rotation testing: the SPV reference ranges for the left and right horizontal nystagmus induced by the left and right rotation around the Z-axis (yaw) were 13.58–75.32°/s and 13.56–74.02°/s, respectively. The SPV reference ranges of the vertical upward and downward nystagmus induced by head elevation and head bowing around the X-axis (pitch) were 13.13–50.21°/s and 11.98–48.04°/s, respectively. For the left/right rotation induced by head tilt around the Y-axis (roll) (from the subject’s perspective), the reference range for normal nystagmus SPV was 10.97–47.02°/s and 12.34–44.35°/s, respectively. The mean values of SPV asymmetry induced by the triaxial rotation around the Z-axis (yaw), X-axis (pitch), and Y-axis (roll) were 11.8 ± 9.1%, 15.1 ± 13.0%, and 10.8 ± 7.8%, respectively, with no statistical significance (*p* = 0.140). The reference ranges of one side were 0, 26.8%, 0, 36.6%, and 0, 23.7%, respectively. The above normal reference ranges can provide a reliable reference for quantitative analysis of the low-frequency damage of different vertical semicircular canals. The asymmetric reference range for SPV of induced nystagmus by the pitch plane rotation is larger than those of nystagmus induced in the yaw and roll planes, which may be related to the effect of receiving the same stimulation in the anterior and posterior semicircular canals. Aw et al. (1998) stimulated the three semicircular canals by caloric tests and found that the induced nystagmus of the lateral semicircular canal was the strongest, and the SPV for the induced nystagmus of the anterior and posterior semicircular canal was 30% and 10%, respectively. The difference between this report and our study results may be due to the different stimulation frequencies of the two methods or the simultaneous stimulation of the two anterior and two posterior semicircular canals in this study.

Modern diagnosis and treatment of vertigo put forward higher requirements for the recording of eye movements. To align with the evolution of big data and intelligence in vertigo diagnosis and treatment, a study (Kattah and Newman-Toker, 2022; Parker et al., 2022) on big data of vertigo telemedicine was conducted in the United States in recent years. However, it was unsuccessful due to the lack of a reliable eye movement recording system. This study, following the description method of nystagmus presented by the Barany Association in 2019 (Eggers et al., 2019), applied a portable 3D-VNG eye mask to carry out the low-frequency manual triaxial rotation testing on each semicircular canal and different semicircular canal combinations. We analyzed the SPV characteristics of induced nystagmus and discussed the application of this method for detecting the lateral semicircular canal. We also evaluated the feasibility of the low-frequency aVOR-induced nystagmus in the vertical semicircular canal. The results of this study show that the manual triaxial rotation testing can be used to evaluate the function of the low-frequency aVOR of the semicircular canals. Specifically, the horizontal nystagmus, induced by rotation around the Z-axis (yaw), can distinguish the damaged side of the left and right lateral semicircular canals. Rotation around the X-axis (pitch) induces vertical nystagmus, which identifies the anterior and posterior semicircular canals. This, in combination with rotation around the Y-axis (roll), identifies the left and right vertical semicircular canals, thereby localizing damage to the vertical semicircular canal. The damage of any one of the vertical semicircular canals can be deduced from the SPV of nystagmus induced by the combinations of “double anterior + double posterior” semicircular canals and “anterior + posterior on each side” semicircular canals by rotating in the pitch plane and roll plane, respectively. For example, nystagmus induced by rotation along the pitch plane is characterized by a smaller SPV for the vertical downward nystagmus compared to that of the vertical upward nystagmus [(LA + RA) < (LP + RP)]. Nystagmus induced by rotation along the roll plane shows a smaller SPV for the right-rotatory nystagmus compared to that of the left-rotatory nystagmus [(RA + RP) < (LA + LP)], suggesting damage to the right anterior (RA) semicircular canal, and vice versa. Similarly, the function of the right anterior/right posterior semicircular canal and the left anterior/left posterior semicircular canal can be evaluated separately based on the SPV characteristics of the nystagmus induced by rotation along the pitch and roll planes (referring to the formula below). The results of our study show the characteristics of nystagmus induced by different semicircular canal combinations, which can help to provide objective support for tracing the injury localization of one or two semicircular canals, especially vertical semicircular canals, and further provide objective quantified big data and machine learning algorithms of AI technology for otogenic disease in the future.

The damage of every one of the vertical semicircular canals can be deduced based on the formula:

1. RA^loss^ ⇒ [(LA + RA^loss^) < (LP + RP)] + [(RA^loss^ + RP) < (LA + LP)];
2. LP^loss^ ⇒ [(LA + RA) < (LP^loss^ + RP)] + [(RA + RP) < (LA + LP^loss^)];
3. LA^loss^ ⇒ [(LA^loss^ + RA) < (LP + RP)] + [(RA + RP) < (LA^loss^ + LP)];
4. RP^loss^ ⇒ [(LA + RA) < (LP + RP^loss^)] + [(RA + RP^loss^) < (LA + LP)].

### 4.2 Mechanism analysis of the low-frequency induced nystagmus of each semicircular canal

The test results showed that turning the head around the Z-axis (yaw) stimulates the left and right lateral semicircular canals, inducing left and right horizontal nystagmus, respectively. Pitching the head around the X-axis (pitch) stimulates the combination of the anterior semicircular canals of both ears and the combination of the posterior semicircular canals of both ears (double-canal mode), inducing vertical downward and upward nystagmus. Rolling the head around the Y-axis (roll) to the left and the right stimulates the anterior and posterior semicircular canals (double-canal mode) of each ear, respectively, inducing significant torsional nystagmus. Given that the lateral semicircular canal is at a physiological angle of 30° to the ground, the torsional nystagmus was accompanied by a mild horizontal nystagmus. The direction and SPV characteristics of nystagmus induced by the triaxial rotation testing were analyzed. The SPV induced by nystagmus in two opposite directions of the same rotation axis was not statistically significant. This suggests that this portable manual triaxial rotation testing can achieve consistent results, namely that an equal amount of induced nystagmus SPV is induced by an equal of stimulation in two opposite directions around the same rotation axis. The test exhibits the characteristic where an equal amount of physiological stimulation induces an equal amount of effect (Figure 2).

### 4.3 Mechanism analysis

4.3.1 When the head rotates to the left around the Z-axis (yaw), the bilateral lateral semicircular canal aligns with the plane of rotation. Consequently, the endolymph fluid in the left lateral (LL) semicircular canal flows to the ampullary crest, producing excitatory stimulation, while, in the right lateral (RL) semicircular canal, it flows away, producing inhibitory stimulation. This excites the left lateral (LL) semicircular canal, activating the left medial rectus (MR) and the right lateral rectus (LR), producing a combined effect to produce a slow-phase eye movement to the right (the fast phase of nystagmus in the opposite direction). Similarly, when the head rotates to the right around the Z-axis (yaw), the bilateral lateral semicircular canal aligns with the rotation plane. Thereafter, the endolymph fluid in the right lateral (RL) semicircular canal flows to the ampulla crest, producing excitatory stimulation, while, in the left lateral (LL) semicircular canal, it flows away, producing inhibitory stimulation. This excites the right lateral (RL) semicircular canal, activating the right medial rectus (MR) and left lateral rectus (LR), producing a combined effect that results in a slow phase movement of the eye to the left (opposite to the nystagmus fast phase).

4.3.2 When the head rotates downward around the X-axis (pitch), a combined equal excitation of both the left anterior (LA) and right anterior (RA) semicircular canals activates bilateral superior rectus (SR) and oblique muscles, causing purely upward slow phases as the torsional components from each canal cancel each other out (purely downward fast phases of nystagmus). Similarly, when the head rotates upward around the X-axis (pitch), a combined equal excitation of both the left posterior (LP) and right posterior (RP) semicircular canals activates bilateral inferior rectus (IR) and oblique muscles, causing purely downward slow phases as the torsional components from each canal cancel each other out (purely upward fast phases of nystagmus). Clinically, bilateral anterior or posterior injuries are rare, but this study induced pure vertical upward or downward nystagmus through the combination of bilateral anterior and bilateral posterior semicircular canals in healthy individuals, further suggesting that pure vertical nystagmus is often caused by damage to the vestibular center.

4.3.3 When the head deflects to the left around the Y-axis (roll), the bilateral anterior and posterior (LA + LP + RA + RP) vertical semicircular canals enter the rotation plane, the endolymph fluid in the left anterior (LA) and posterior (LP) vertical semicircular canals flows away from the ampullary crest, producing a joint excitatory stimulus. The endolymph fluid in the right anterior (RA) and posterior (RP) vertical semicircular canal flows toward the ampullary crest, producing a combined inhibitory stimulus. This excites the left anterior (LA) canal, activating the right inferior oblique (IO) (large), right superior rectus (SR), left superior oblique (SO), and left superior rectus (SR) (large). Excitation of the left posterior (LP) semicircular canal excitation activates the right inferior oblique muscle (IO), right inferior rectus (IR) (large), left superior oblique muscle (SO) (large), and left inferior rectus (IR). The vertical components of the left vertical semicircular canal up and down cancel each other out, resulting in a slow phase movement of the upper pole of the eye twisting to the right ear (from the subject’s perspective). This produces nystagmus in which the upper pole of the eye twists to the left ear (from the subject’s perspective) or purely clockwise nystagmus (from the physician’s perspective). Similarly, when the head deflects to the right around the Y-axis (roll), both the bilateral anterior and posterior (LA + LP + RA + RP) vertical semicircular canals align with the rotation plane. The endolymph fluid in the right anterior (RA) and posterior (RP) vertical semicircular canal flows away from the ampullary crest, producing a joint excitatory stimulus. Conversely, in the left anterior (LA) and posterior (LP) vertical semicircular canal, the endolymph fluid flows toward the ampullary crest, producing a joint inhibitory stimulus. Excitation of the right anterior (RA) semicircular canal activates the left inferior oblique (IO) (large), left superior rectus (SR), right superior oblique (SO), and right superior rectus (SR) (large). Excitation of the right posterior (RP) semicircular canal activates the left inferior oblique (IO), left inferior rectus (IR) (large), right superior oblique (SO) (large), and right inferior rectus (IR). Thereafter, the vertical components of the right vertical semicircular canal up and down cancel each other out, resulting in a slow phase movement of the upper pole of the eye to the left ear (from the subject’s perspective). This produces nystagmus in which the upper part of the eye moves to the right ear (from the subject’s perspective) or pure counterclockwise nystagmus (from the physician’s perspective).

### 4.4 Limitations and further research

This study’s observations were limited to healthy young people; further research is needed to extend these findings to other age groups. Although rotation in the left anterior/right posterior semicircular canal conjugate plane is the most effective stimulation of the vertical canal, this study did not adopt this stimulation method due to the effect of pupillary exposure on the 3D nystagmus recording. Further research should focus on overcoming these limitations and improving the 3D recording of nystagmus in different head positions. Multi-frequency, multi-axial vertical semicircular canal induction test represents a promising direction for future research. Portable binocular 3D-VNG, which may be facilitated in bedside examination or as an in-home vestibular event monitor to capture intermittent events for later analysis (Young et al., 2019), makes it possible for telemedicine and AI diagnosis based on bid data in the future.

## 5 Conclusion

In summary, the manual triaxial rotation testing, facilitated by a portable 3D-VNG eye mask, effectively induces the characteristic nystagmus, and enables the evaluation of the vertical semicircular canal’s low-frequency aVOR function. This method is convenient, efficient, and practical. Moreover, it elucidates the three-dimensional characteristics of nystagmus induced by different semicircular canal combinations in healthy young people. The reference range for the SPV of the vertical semicircular canal-induced nystagmus and its asymmetric reference range in healthy young people were preliminarily obtained. These findings provide a basis for the traceability of nystagmus in patients with otogenic vertigo and pave the way for further research on the mechanism of semicircular canal-induced nystagmus, baseline evaluation of various otogenic vertigo diseases, precision diagnosis, and treatment rehabilitation as well as big data telemedicine.

## Data availability statement

The original contributions presented in the study are included in the article/supplementary material, further inquiries can be directed to the corresponding author/s.

## Ethics statement

The studies involving humans were approved by the Ethics Committee of Tianjin First Central Hospital, China. The studies were conducted in accordance with the local legislation and institutional requirements. Written informed consent for participation in this study was provided by the participants’ legal guardians/next of kin.

## Author contributions

XH: Formal analysis, Investigation, Methodology, Writing – original draft, Writing – review & editing, Software. XZ: Data curation, Formal analysis, Writing – review & editing. QD: Investigation, Writing – review & editing. SL: Investigation, Writing – review & editing. QL: Investigation, Writing – review & editing. CW: Investigation, Writing – review & editing. WW: Conceptualization, Funding acquisition, Methodology, Project administration, Resources, Supervision, Writing – review & editing. TC: Conceptualization, Funding acquisition, Methodology, Project administration, Resources, Supervision, Writing – review & editing.
